# Global spatiotemporally continuous MODIS land surface temperature dataset

**DOI:** 10.1038/s41597-022-01214-8

**Published:** 2022-04-01

**Authors:** Pei Yu, Tianjie Zhao, Jiancheng Shi, Youhua Ran, Li Jia, Dabin Ji, Huazhu Xue

**Affiliations:** 1 School of Surveying and Land Information Engineering, Henan Polytechnic University, Jiaozuo, China; 2 State Key Laboratory of Remote Sensing Science, Aerospace Information Research Institute, Chinese Academy of Sciences, Beijing, China; 3 National Space Science Center, Chinese Academy of Sciences, Beijing, China; 4 Northwest Institute of Eco-Environment and Resources, Chinese Academy of Sciences, Lanzhou, China

**Keywords:** Hydrology, Hydrology

## Abstract

Land surface temperature (LST) plays a critical role in land surface processes. However, as one of the effective means for obtaining global LST observations, remote sensing observations are inherently affected by cloud cover, resulting in varying degrees of missing data in satellite-derived LST products. Here, we propose a solution. First, the data interpolating empirical orthogonal functions (DINEOF) method is used to reconstruct invalid LSTs in cloud-contaminated areas into ideal, clear-sky LSTs. Then, a cumulative distribution function (CDF) matching-based method is developed to correct the ideal, clear-sky LSTs to the real LSTs. Experimental results prove that this method can effectively reconstruct missing LST data and guarantee acceptable accuracy in most regions of the world, with RMSEs of 1–2 K and R values of 0.820–0.996 under ideal, clear-sky conditions and RMSEs of 4–7 K and R values of 0.811–0.933 under all weather conditions. Finally, a spatiotemporally continuous MODIS LST dataset at 0.05° latitude/longitude grids is produced based on the above method.

## Abstract (summary)

**Table:** 

Measurement(s)	land surface temperature
Technology Type(s)	satellite imaging
Sample Characteristic - Environment	planetary surface
Sample Characteristic - Location	global

## Background & Summary

Land surface temperature (LST) plays a critical role in the study of the physical and biological processes of the Earth’s surface at global and regional scales^1^ and is also closely related to changes in the variables that characterize the key states of the Earth’s systems, such as water vapor content, soil moisture, evapotranspiration statuses and land surface freeze-thaw statuses^2–8^. Therefore, LST is widely used in the research fields of ecology, environmental studies, hydrology, meteorology and climate studies, and agricultural production^9–14^. At present, LSTs can be mainly derived through three approaches, including field or *in-situ* measurements^15^, satellite observations, and model simulations^16^. Field and *in-situ* measurements are not easily affected by weather or other factors, and LSTs can be obtained continuously over time. However, the usefulness of such data is poor when the field stations are sparsely distributed^17^. Most model reanalysis datasets, such as the Modern-Era Retrospective Analysis for Research and Applications (MERRA) dataset, National Center for Environmental Prediction (NCEP) products, and the European Center for Medium-Range Weather Forecasts (ECMWF) Reanalysis product ERA-Interim^18^, can provide spatiotemporally continuous LSTs at a global scale. However, these reanalysis datasets are usually output with coarse resolutions, and the effects of surface properties on LSTs are only roughly considered in these numerical models, which are unable to meet the requirements of many applications, which demand LSTs with finer resolution. Given these facts, satellite remote sensing technology has become popularly for observing LSTs with acceptable temporal and spatial resolutions over the entire globe^19^.

With the development of remote sensing technology, there are currently many sensors that can provide LST products, such as EOS/MODIS, NOAA/AVHRR and FY/VISSR. The validation accuracy of MODIS LST products (MYD11/MOD11) can reach 1 K under clear-sky conditions^20^. Such satellite-based LST products can also derive many other data products, such as lake surface water temperatures (LSWTs)^21,22^, which are widely used in various studies. At present, remote sensing LST products are often obtained via retrieval from the land surface and atmospheric parameters (e.g., transmittance and emissivity) measured by satellites using a variety of split-window algorithms^23,24^. However, this category of algorithms works only under clear-sky conditions; thus, when affected by clouds or other atmospheric disturbances, an MODIS LST product may possess large proportions of missing data^25^, which greatly limits the application of MODIS LST products. To overcome this problem, a variety of LST reconstruction techniques have been developed. A category of methods to reconstruct LSTs contaminated by clouds has been developed by using spatially, temporally or spatiotemporally neighboring available clear-sky LSTs (i.e., temporal or spatial interpolation)^26,27^. Another category of approaches not only uses neighboring clear-sky LSTs but also combines auxiliary variables such as latitude, longitude, elevation and NDVI to reconstruct missing LSTs^28–30^.

However, the abovementioned methods can only reconstruct the missing LST values under ideal, clear-sky conditions but cannot construct real LSTs under cloudy sky conditions. In general, clouds reduce incoming shortwave radiation during the daytime by blocking the sun and increase the downward longwave radiation during the nighttime^31^. Therefore, there is usually a deviation between a cloud-covered LST and a cloud-free LST. To solve the above problems, some physical modeling approaches, such as surface energy balance (SEB) theory, have been developed to reconstruct real LSTs under cloudy sky conditions^32,33^, and this category of methods was mainly accomplished by establishing relationships between the LSTs of cloudy pixels and their neighboring clear pixels. However, some of these methods need to calculate regional parameters from ground-based measurements, and some are not effective when larger areas of data are missing, so they are difficult to apply to LST reconstruction at large scales from global perspectives.

Here, with the data interpolating empirical orthogonal functions (DINEOF) method and the ECMWF ERA5-Land climate reanalysis dataset, we develop a simple and effective method for global cloud-contaminated LST reconstruction and provide a global spatiotemporally continuous MODIS LST dataset from 2002 to 2020 with a spatial resolution of 0.05°.

## Methods

**Fig. 1:**
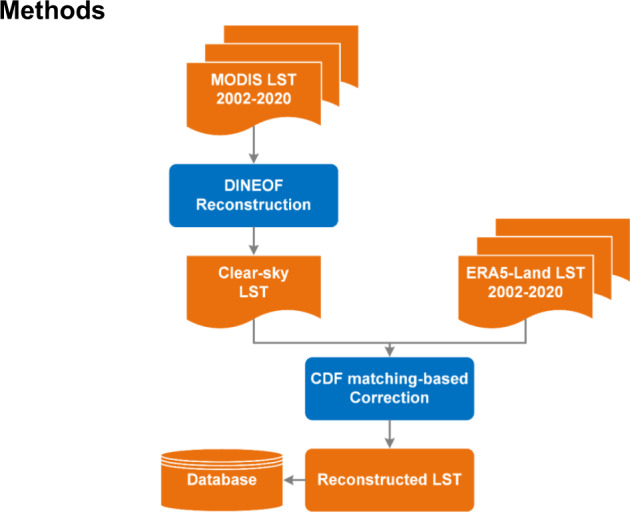
Flowchart of the two-step framework developed for LST reconstruction.

As shown in the flowchart in Fig. 1, the first step is to use the DINEOF reconstruction process to obtain the ideal clear-sky LSTs for cloud-contaminated areas. In the second step, the ideal clear-sky LSTs are corrected to the real LSTs by a cumulative distribution function (CDF) matching-based method with LSTs from the ERA5-Land climate reanalysis data.

### Satellite and climate reanalysis data

**Fig. 2:**
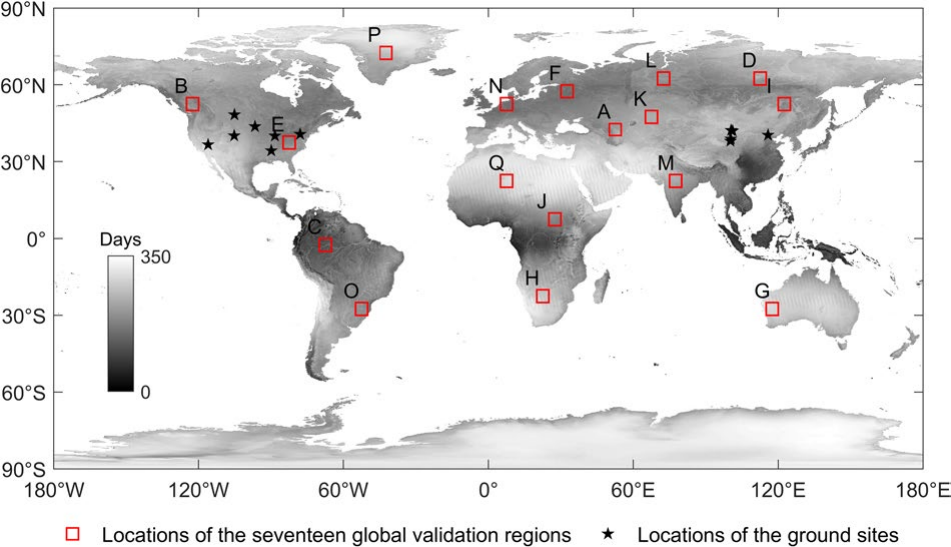
Locations of the validation areas and the ground sites. The grayscale image represents the average number of clear-sky days per year from 2002 to 2020, the red rectangles represent the locations of the seventeen global validation regions, and the black pentagrams represent the locations of the ground sites.

We use the level-3 daily global LST products (MOD11C1/MYD11C1, Collection 6) provided by two polar-orbiting sun-synchronous satellites, Terra and Aqua (10:30 AM/PM and 1:30 AM/PM local solar time, respectively), from the NASA Earth Observing System, which provide temperature and emissivity values at 0.05° latitude/longitude climate model grids (CMGs)^34^. It has been verified that the accuracy of the improved MODIS collection 6 (C6) is better than that of C5^35^, and the errors are less than 1 K at most sites on a uniform surface. The temperature and emissivity values in MOD11C1 and MYD11C1 are derived by reprojection and by averaging the values in the daily MODIS LST/E product (MOD11B1/MYD11B1) at 6-km equal-area grids in the sinusoidal projection. The LST values aggregated to 6-km grids from those retrieved by the generalized split-window algorithm are used to supplement the LSTs retrieved by the day/night LST algorithm at grids where there are no valid pairs of day and night observations (usually in high-latitude regions). Figure 2 shows the global annual average of the number of days with cloud-free MODIS LST data from 2002 to 2020.

In this work, we also include the climate reanalysis data from ERA5-Land’s skin temperature data (hereinafter referred to as ERA5-Land LST data) based on the HTESSEL model^36^. The ERA5-Land LST is the theoretical temperature that is required to satisfy the surface energy balance, which has the same physical meaning as the MODIS LST. ERA5-Land was produced by replaying the land component of the ECMWF ERA5-Land climate reanalysis dataset^37^. The temporal and spatial resolutions of ERA5-Land make this dataset very useful for all kinds of land surface applications. ERA5-Land provides data at 0.1° regular latitude/longitude grids with an hourly output frequency, and users can freely obtain data from the Copernicus Climate Change Service (C3S) Climate Data Store (CDS) for 1981 to the present. Johannsen *et al*. and Wang evaluated the ERA5-Land LST data using other satellite-derived LSTs and *in-situ* measurements, respectively, and the results proved that the ERA5-Land LST dataset has good usability^38,39^.

### Data interpolating empirical orthogonal functions (DINEOF) reconstruction method

The DINEOF method is a self-consistent method that was first developed by Beckers *et al*.^40^ to reconstruct incomplete oceanographic datasets based on the empirical orthogonal functions (EOF) method. Subsequently, Alvera-Azcárate *et al*. used the Lanczos method^41^ to improve the DINEOF approach so that it could be used to reconstruct datasets with large amounts of missing data^42^ and reconstruct the sea surface temperature (SST) of the Adriatic Sea. Recently, Zhou *et al*. reconstructed the LST of Ali on the Tibetan Plateau from 2002 to 2016 by using the DINEOF method^16^ and proved that the DINEOF method could accurately recover missing LSTs. The DINEOF method is an automatic, parameter-free, and self-consistent gap-filling method that is different from the traditional methods used in geoscience. It does not require any a priori knowledge of the original data and has high computational efficiency. The principle of the DINEOF method when applied to LST reconstruction can be explained as follows:

Assuming that *LST*_*matrix*_ is the initial matrix, which contains the observations and some unknown values corresponding to the missing data, the dimensions of *LST*_*matrix*_ are *s* × *t* (*s* is the spatial dimension and *t* is the temporal dimension). The DINEOF method infers missing data based on the empirical orthogonal functions (EOFs) of the data. Here, we can use the singular value decomposition (SVD) method to obtain the EOFs of *LST*_*matrix*_:

1 $${LST}_{matrix}=U\sum {V}^{T}$$

where *U* is the spatial EOF of *LST*_*matrix*_, *V* represents the temporal EOF of *LST*_*matrix*_, and Σ contains the singular values of *LST*_*matrix*_. Then, the missing data *LST*_*ij*_ in *LST*_*matrix*_ can be accurately reconstructed via Eq. (2).

2 $${LST}_{ij}={\sum }_{{\rm{n}}={\rm{1}}}^{k}{\rho }_{n}{\left({u}_{n}\right)}_{i}{\left({v}_{n}^{T}\right)}_{j}$$

*u*_*n*_ and *v*_*n*_ are the nth columns of *U* and *V*, respectively, corresponding to the singular value *ρ*_*n*_. The specific technical process of reconstructing the missing LST data by using the DINEOF method is as follows:

- Randomly select some valid observations from the original dataset as the validation dataset to prepare for subsequent cross validation and remove the values of the validation pixels from the original dataset.
- Fill the missing data points with zeros to obtain a first guess of the missing data.
- Apply EOF decomposition to the matrix, extract the first k EOFs to reconstruct the original matrix, and replace the missing values by the *LST*_*ij*_ obtained with the EOFs. Repeat this process until convergence is reached.
- Then, repeat the above process by increasing the number of computed EOFs from k = 1, 2, …, *k*_*optimal*_ until the cross-validation accuracy exceeds the present value, and then determine *k*_*optimal*_ as the optimal number of EOFs.
- Add the cross-validation dataset to the original dataset and use the optimal number of EOFs *k*_*optimal*_ to repeat the whole procedure for the original dataset. Then, the missing data can be reconstructed accurately.

**Fig. 3:**
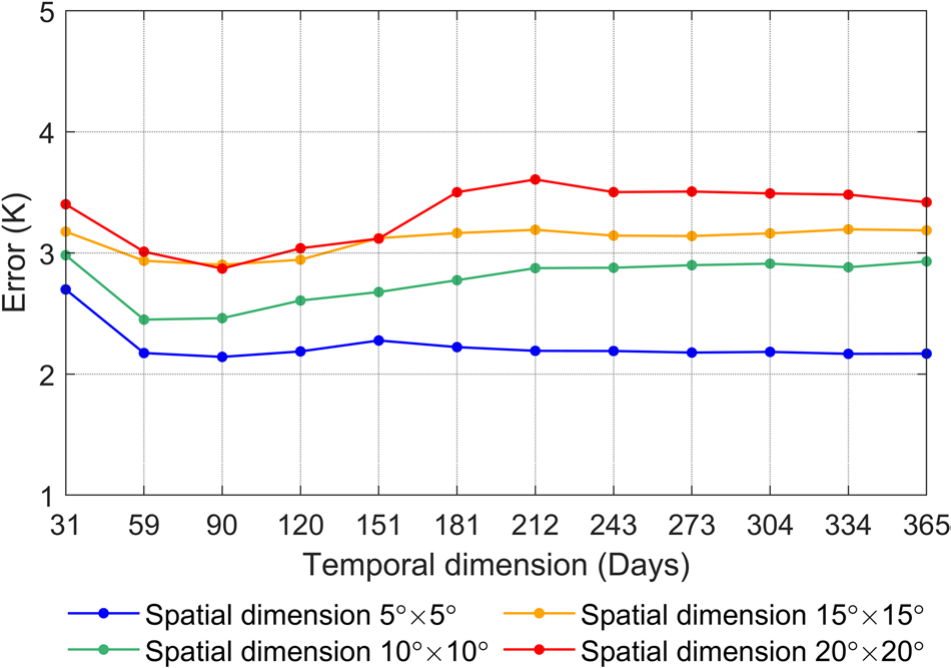
Variations in the DINEOF reconstruction error with different spatial and temporal LST dimensions.

The spatial dimension *s* and the temporal dimension *t* of *LST*_*matrix*_ are the key factors that affect the reconstruction accuracy of the DINEOF method. The variations in the DINEOF reconstruction error with different spatial and temporal LST dimensions are shown in Fig. 3. As the spatial dimension increases, the error of an LST reconstructed with the DINEOF method also increases significantly. The reason for this is probably that with the increasing size of the spatial dimension, the number of missing points becomes larger, and it is difficult to find the optimal number of EOFs for all missing points, which may affect the final reconstruction accuracy. In contrast, as the temporal LST dimension increases, the error of a DINEOF-reconstructed LST gradually decreases and becomes stable. Considering the computational efficiency and reconstruction accuracy, we finally determine that the spatial and temporal dimensions for reconstructing the global LST with the DINEOF method are 5° × 5° and 365 days (a whole year), respectively, and we then utilize a sliding window at the global scale and use the average as the final outputs for more robust LST estimation to produce the global spatiotemporally continuous ideal, clear-sky MODIS LST dataset with a 0.05° spatial resolution from 2002 to 2020.

### Cumulative distribution function (CDF) matching-based correction

Since clouds may reduce the solar radiation that reaches the surface, causing an ideal, clear-sky LST to deviate from the LST obtained under cloudy sky conditions, it is necessary to correct the ideal, clear-sky LSTs reconstructed by the DINEOF method to the real LSTs under cloudy sky conditions.

The cumulative distribution function (CDF), also called the distribution function, is the integral of the probability density function, and it can completely describe the probability distribution of a real random variable *X*. The cumulative distribution function matching (CDF matching) method was first proposed by Calheiros *et al*.^43^ and then used to calibrate radar data, remote sensing observations, and precipitation data^44,45^. The CDF matching method utilizes a certain kind of reliable data to correct and fuse remote sensing data from other sources, which can improve the spatial or temporal resolution and the accuracy of the remote sensing data. The CDF matching method does not change the original relative change pattern of remote sensing data^46^, and it can adjust the overall range of data values to be close to the true value range. ERA5-Land is a reanalysis dataset that combines model data with observations from across the world by using the laws of physics. ERA5-Land LST is an all-weather LST dataset, including clear sky and cloudy sky conditions. Taking these LSTs into account, a method based on CDF matching is proposed here to use the ERA5-Land LST dataset to correct the clear-sky LSTs reconstructed by the DINEOF method to the real LSTs under cloudy sky conditions. The basic principle is as follows.

The LST at a specific time and date can be regarded as consisting of two parts: one is the long-term mean of the temperature at that time (climatological temperature), and the other is the deviation from that climatological temperature due to the weather (anomaly temperature)^17^. First, we calculate the climatological temperatures of the ideal, clear-sky satellite and the -ERA5-Land LSTs by using the reconstructed ideal clear-sky MODIS LST and ERA5-land LST data from 2002 to 2020:

3 $${LST}_{clim}^{clear \mbox{-} sky}\left(i\right)=\overline{{LST}^{clear \mbox{-} sky}\left(i\right)}$$

4 $${T}_{{\rm{clim}}}\left(i\right)=\overline{T\left(i\right)}$$

where ${LST}_{clim}^{clear \mbox{-} sky}\left(i\right)$ is the climatological temperature of the ideal, clear-sky satellite on day *i* of the year, $\overline{{LST}_{clim}^{clear \mbox{-} sky}\left(i\right)}$ is the mean of the reconstructed ideal clear-sky MODIS LST on day *i* of each year from 2002 to 2020, ${T}_{clim}\left(i\right)$ is the climatological temperature of the ERA5-Land LST on day *i* of the year, and $\overline{T\left(i\right)}$ is the mean of the ERA5-Land LST on day *i* of each year from 2002 to 2020.

Considering the influence of clouds, the climatological temperatures of the ideal clear-sky satellite may deviate from the real climatological temperatures under cloudy sky conditions. Here, the climatological temperature of the ideal clear-sky satellite is corrected to the real climatological temperatures under cloudy sky conditions by Eq. (5):

5 $${LST}_{clim}^{cloudy}(i)={LST}_{clim}^{clear \mbox{-} sky}(i)-\bar{({LST}_{clim}^{clear \mbox{-} sky}}-\bar{{T}_{{\rm{clim}}}})$$

where ${LST}_{clim}^{cloudy}\left(i\right)$ is the climatological temperature of the real satellite under cloudy sky conditions, $\bar{{LST}_{clim}^{clear \mbox{-} sky}}$ is the mean annual climatological temperature of the ideal clear-sky satellite, and $\bar{{T}_{clim}}$ is the mean annual climatological temperature of the ERA5-Land LST. Then, the anomaly temperature of the reconstructed ideal clear-sky MODIS LST and the ERA5-Land LST can be calculated:

6 $${LST}_{anom}^{clear \mbox{-} sky}\left(i\right)={LST}^{clear \mbox{-} sky}\left(i\right)-{LST}_{clim}^{cloudy}\left(i\right)$$

7 $${T}_{anom}\left(i\right)=T\left(i\right)-{T}_{clim}\left(i\right)$$

where ${LST}_{anom}^{clear \mbox{-} sky}\left(i\right)$ is the anomaly temperature of the reconstructed ideal, clear-sky MODIS LST, ${LST}^{clear \mbox{-} sky}\left(i\right)$ is the reconstructed ideal, clear-sky LST, ${T}_{anom}\left(i\right)$ is the anomaly temperature of the ERA5-Land LST, and *T*(*i*) is the ERA5-Land LST, which corresponds to the reconstructed ideal, clear-sky MODIS LST.

Since ERA5-Land LST considers the influence of clouds and other factors on LST changes, we can propose a hypothesis: the anomalous temperatures obtained from satellite estimates under cloudy sky conditions and those of the ERA5-Land LST dataset should have the same cumulative distribution function:

8 $${\rm{CDF}}\left({LST}_{anom}^{cloudy}\right)=CDF\left({T}_{anom}\right)$$

Therefore, the anomaly temperature of the reconstructed ideal, clear-sky MODIS LST ${LST}_{anom}^{clear \mbox{-} sky}$ can be corrected to that of the real satellite under cloudy sky conditions ${LST}_{anom}^{cloudy}$ through the CDF matching method, and then the real satellite LST under cloudy sky conditions ${LST}^{cloudy}$ after correction can be obtained:

9 $${LST}^{cloudy}\left(i\right)={LST}_{clim}^{cloudy}\left(i\right)+{LST}_{anom}^{cloudy}\left(i\right)$$

Next, we correct the reconstructed ideal, clear-sky MODIS LSTs to the real LSTs under cloudy sky conditions with the CDF matching method and output one set of global spatiotemporally continuous all-weather dynamic (including clear-sky and cloudy-sky) MODIS LST products with a 0.05° spatial resolution from 2002 to 2020. The actual clear-sky satellite observations in the original MODIS LST products are retained whenever they are available.

### Statistical metrics

Here, six statistical metrics are used to quantify the reconstruction performance: the Bias, root mean square error (RMSE), unbiased root mean square error (ubRMSE), root mean square difference (RMSD), unbiased root mean square difference (ubRMSD), and Pearson correlation coefficient ®, which are defined as follows:

10 $${\rm{Bias\; =}}\frac{1}{n}{\sum }_{i=1}^{n}\left({LST}_{ri}-{LST}_{oi}\right)$$

11 $${\rm{RMSE}}=\sqrt{\frac{1}{n}{\sum }_{i=1}^{1}{\left({LST}_{ri}-{LST}_{oi}\right)}^{2}}$$

12 $${\rm{ubRMSE}}=\sqrt{{{\rm{RMSE}}}^{2}-{{\rm{Bias}}}^{2}}$$

13 $${\rm{RMSD}}=\sqrt{\frac{1}{n}{\sum }_{i=1}^{1}{\left({LST}_{ri}-{LST}_{oi}\right)}^{2}}$$

14 $${\rm{ubRMSD}}=\sqrt{{{\rm{RMSD}}}^{2}-{{\rm{Bias}}}^{2}}$$

15 $${\rm{R}}=\frac{{\sum }_{i=1}^{n}\left({LST}_{ri}-\bar{{LST}_{ri}}\right)\left({LST}_{oi}-\bar{{LST}_{oi}}\right)}{\sqrt{{\sum }_{i=1}^{n}{\left({LST}_{ri}-\bar{{LST}_{ri}}\right)}^{2}}\sqrt{{\sum }_{i=1}^{n}{\left({LST}_{oi}-\bar{{LST}_{oi}}\right)}^{2}}}$$

where *n* is the total number of samples involved in the comparison, *LST*_*ri*_ is the reconstructed LST, and *LST*_*oi*_ represents the LST corresponding to a different validation process, which can be the original satellite-derived LST, the ground-measured LST or the ERA5-Land LST. $\bar{{LST}_{ri}}$ and $\bar{{LST}_{oi}}$ are the means of *LST*_*ri*_ and *LST*_*oi*_, respectively.

## Data Records

The global spatiotemporally continuous MODIS land surface temperature dataset in this study is hosted at the National Tibetan Plateau/Third Pole Environment Data Center^47^ (10.11888/Meteoro.tpdc.271663) with two sets of files: (a) a global spatiotemporally continuous ideal, clear-sky MODIS LST dataset with a 0.05° spatial resolution from 2002 to 2020 and (b) global spatiotemporally continuous all-weather dynamic MODIS LST products with 0.05° spatial resolutions from 2002 to 2020.

All the data are stored in the hdf5 format, and the file names of the ideal, clear-sky MODIS LST data follow this regulation: <MOD11C1(MYD11C1)_YYYYDDD_Clear-sky >.h5, where MOD11C1(MYD11C1) represents the MODIS LST product of the Terra (Aqua) polar-orbiting NASA sun-synchronous satellite, <YYYY> is the year, <DDD> represents the day of the year, and <Clear-sky> indicates that the data are ideal, clear-sky MODIS LST data. The file names of the all-weather dynamic MODIS LST data follow another regulation: <MOD11C1(MYD11C1)_YYYYDDD_All-weather >.h5, where <All-weather >indicates that the data are all-weather dynamic MODIS LST data. The files of each year (2002–2020) are stored in a separate folder.

For the reconstructed ideal, clear-sky MODIS LST data and the all-weather dynamic MODIS LST data, there are all 10 scientific datasets (SDSs) in the daily file, including LST_Day_CMG, QC_Day, Day_view_time, Day_view_angl, LST_Day_filled_flag, LST_Night_CMG, QC_Night, Night_view_time, Night_view_angl, and LST_Night_filled_flag; the QC_Day, Day_view_time, Day_view_angl, QC_Night, Night_view_time and Night_view_angl SDSs are from the original MODIS LST product. Their detailed information is shown in Table 1.

**Table 1:** Detailed information about the SDSs in the global spatiotemporally continuous MODIS LST product.

SDS Name	Long Name	Number Type	Unit	Fill Value	Scale Factor	Added Offset
LST_Day_CMG	Daily daytime reconstructed CMG Land surface temperature	uint16	K	0	0.02	0
QC_Day	Quality control for the daytime LSTs	uint8	none	0	none	none
Day_view_time	Time of day of the LST observation (UTC)	uint8	hrs	0	0.2	0
Day_view_angl	View zenith angle of the daytime land surface temperature	uint8	deg	255	1.0	−65.0
LST_Day_filled_flag	Flags indicating original LST_Day_CMG data or filled data	uint8	none	0	none	none
LST_Night_CMG	Daily nighttime reconstructed CMG land surface temperature	uint16	K	0	0.02	0
QC_Night	Quality control for the nighttime LSTs	uint8	none	0	none	none
Night_view_time	Time of night for the LST observation (UTC)	uint8	hrs	0	0.2	0
Night_view_angl	View zenith angle of the nighttime land surface temperature	uint8	deg	255	1.0	−65.0
LST_Night_filled_flag	Flags indicating original LST_Night_CMG data or filled data	uint8	none	0	none	none

## Technical Validation

### Performance under different land cover types

**Fig. 4:**
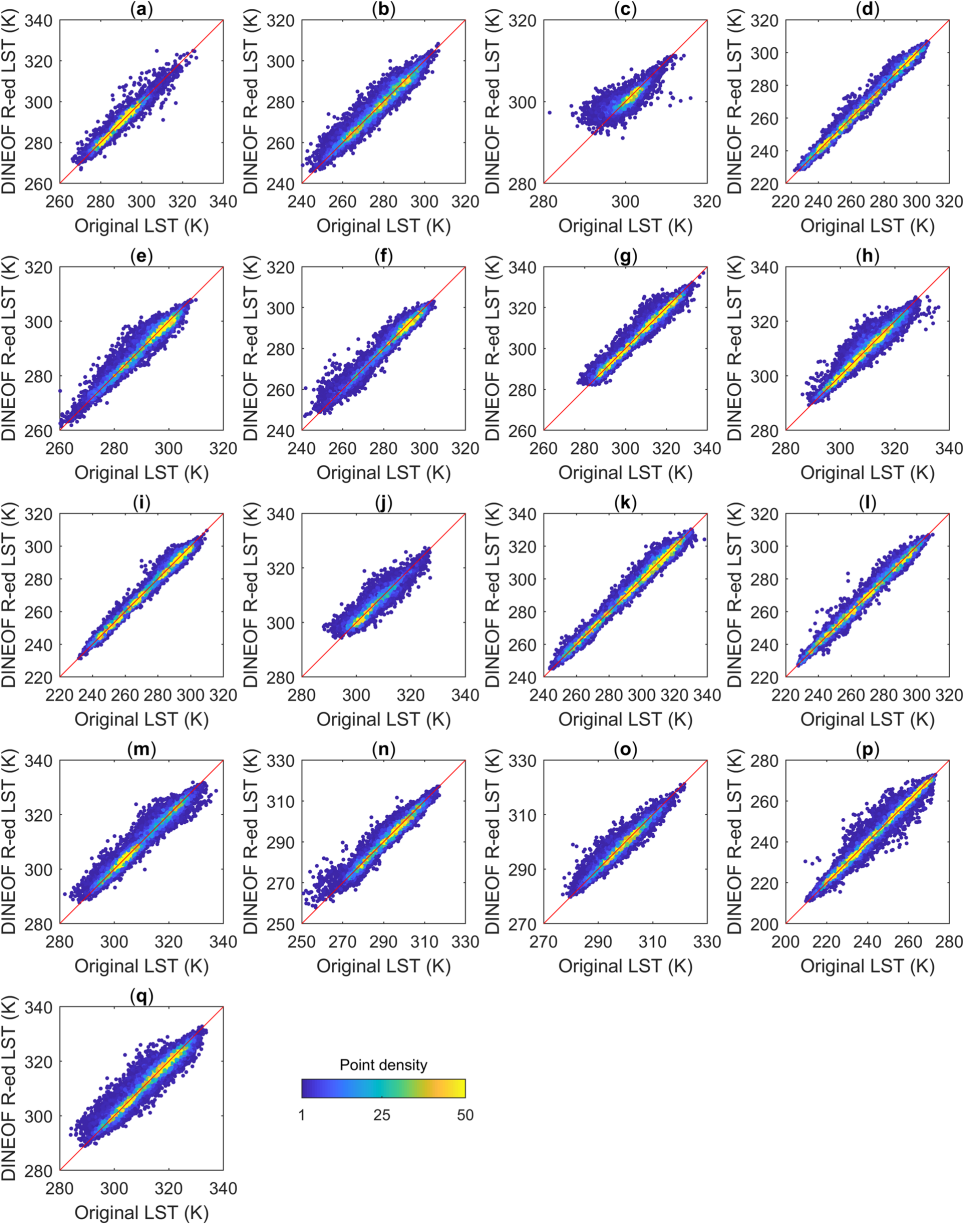
Scatter plots of the DINEOF-reconstructed LSTs (abbreviated in the figure as DINEOF R-ed LST) and the original LSTs under synthetic clouds for different validation regions. (**a**–**q**) represent validation regions a-q, respectively. The red lines are the 1:1 lines, and the colors of scatters represents their point densities.

We randomly selected seventeen validation regions (red rectangles in Fig. 2) around the world according to their land cover types (based on the International Geosphere-Biosphere Programme (IGBP) classification scheme). The DINEOF ideal, clear-sky LST reconstruction method using cloud-free MODIS LST pixels (MYD11C1) was evaluated in these selected regions, and the spatial range of each of these selected regions was 5° × 5°. We eliminated the known clear-sky LSTs and used the DINEOF method to fill in the missing data; then, we compared the errors between the filled data and the original MODIS data. For the above areas with different land cover types, the comparison statistics (overall performance metrics) of the DINEOF method with respect to reconstructing ideal, clear-sky LSTs under synthetic clouds are shown in Fig. 4 and Table 2. Overall, the DINEOF method showed good performance, with an average R of 0.971, a minimum Bias of −0.001 K, a maximum Bias of 0.049 K, and an RMSE between 1.436 K and 2.688 K. The minimum Bias of −0.001 K was achieved in the closed shrubland, while the water areas had the smallest RMSE of 1.436 K, and the deciduous needleleaf forest had the highest correlation of 0.996. The worst correlation coefficient of 0.820 was found for the evergreen broadleaf forest due to it possessing the smallest temperature range throughout the year. From the above statistical metrics, it is shown that the DINEOF method generally effectively reconstructs missing LST information under all land cover types.

**Table 2:** Overall performance of the DINEOF method for different land cover types.

Validation region (land cover type)	Bias (K)	RMSE (K)	R
A (Water)	0.016	1.436	0.981
B (evergreen needleleaf forest)	0.049	2.688	0.976
C (evergreen broadleaf forest)	0.038	1.615	0.820
D (deciduous needleleaf forest)	0.032	1.889	0.996
E (deciduous broadleaf forest)	0.010	1.907	0.976
F (mixed forest)	0.018	2.158	0.983
G (closed shrubland)	−0.001	1.784	0.989
H (open shrubland)	0.017	1.756	0.975
I (woody savanna)	0.009	1.982	0.994
J (savanna)	0.040	2.018	0.944
K (grassland)	−0.006	2.196	0.994
L (permanent wetland)	−0.007	2.119	0.994
M (cropland)	0.020	1.858	0.982
N (urban and built-up)	−0.029	2.347	0.976
O (cropland/natural vegetation mosaic)	−0.031	1.679	0.970
P (snow and ice)	−0.019	2.003	0.991
Q (barren or sparsely vegetated)	0.019	2.301	0.969

### Validation against *in-situ* measurements

To further evaluate the reliability of the CDF matching-based correction method, we compared the original satellite-derived LSTs and reconstructed LSTs with the *in-situ* measured data at 12 ground sites (black pentagrams in Fig. 2). As shown in Fig. 2, among all sites, BON, FPK, GWN, TBL, DRA, PSU and SXF are located in the United States. They are part of the SURFRAD Network, which was established in 1993 with the primary objective of supporting climate research with accurate, continuous, long-term surface radiation budget measurements over the United States^48^. DM^49^, DES^50^, SDQ^51^, YK^52^ and HL^53^ are located in China, where DM, DES, SDQ, and YK are part of the Heihe integrated observatory network^54–56^ and the HL site is part of the multiscale surface flux and meteorological element observation network in the Hai River Basin^57,58^. These sites provided infrared surface radiation values and meteorological observations, such as air temperatures and wind speeds, with a 3-min SURFRAD Network output frequency before 2009 and a 1-min output frequency after 2009. The data output frequency of the Heihe integrated observatory network and the multiscale surface flux and meteorological element observation network in the Hai River Basin was “every 10 minutes”. Then, the *in-situ* LST could be retrieved based on the measured upwelling and downwelling longwave radiation values^59^ calculated by Eq. (16), and this LST was used to approximately represent the ground-truth LST:

16 $${LST}_{in \mbox{-} situ}={\left[\frac{ULR-\left(1-{\varepsilon }_{b}\right)DLR}{{\varepsilon }_{b}\sigma }\right]}^{{\rm{0}}{\rm{.25}}}$$

where *ULR* and *DLR* are the measured upwelling and downwelling longwave radiation, respectively, *ε*_*b*_ is the broadband surface emissivity (BBE) at the location of the ground site, and *σ* is the Stefan-Boltzmann constant (5.67 × 10^−8^ W*m*^−2^*K*^−4^). The details of the twelve ground sites are shown in Table 3.

**Table 3:** Detailed information about the twelve *in-situ* measurement sites.

Site name	Latitude	Longitude	Elevation	Surface type at station
Bondville (BON)	40.051° N	88.373° W	213 m	Grassland
Fort Peck (FPK)	48.308° N	105.102° W	636 m	Grassland
Goodwin Creek (GWN)	34.255° N	89.873° W	96 m	Grassland
Table Mountain (TBL)	40.126° N	105.238° W	1692 m	Sparse grassland
Desert Rock (DRA)	36.623° N	116.020° W	1004 m	Arid shrubland
Penn State U. (PSU)	40.720° N	77.931° W	373 m	Cropland
Sioux Falls (SXF)	43.734° N	96.623° W	483 m	Grassland
Daman (DM)	38.855° N	100.372° E	1556 m	Cropland
Desert (DES)	42.113° N	100.987° E	1054 m	Desert
Sidaoqiao (SDQ)	42.001° N	101.137° E	873 m	Shrubland
Yakou (YK)	38.014° N	100.242° E	4148 m	Alpine meadow
Huailai (HL)	40.357° N	115.792° E	480 m	Cropland

**Fig. 5:**
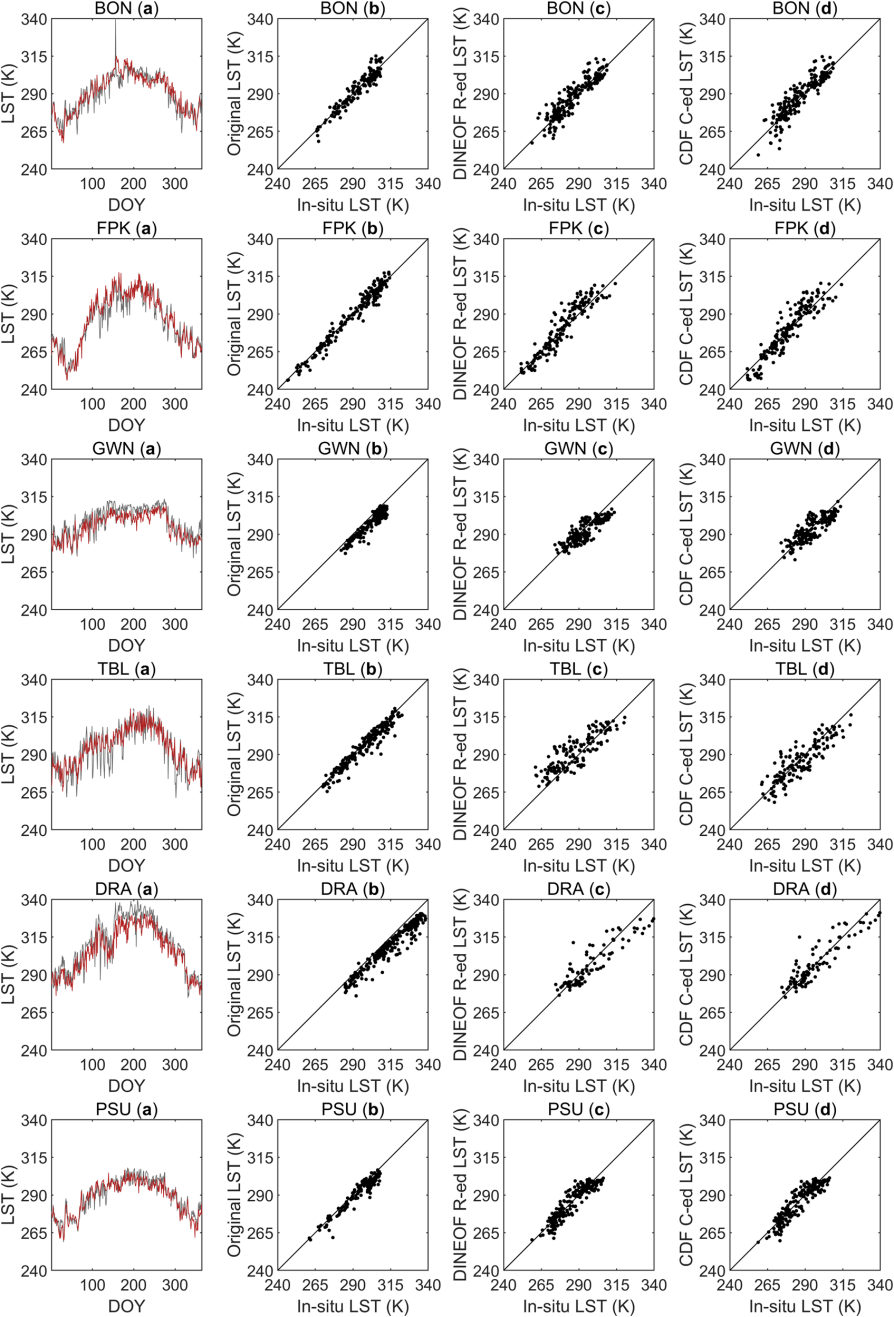
Comparison between the original satellite-derived LSTs and the reconstructed LSTs with the *in-situ* measured LSTs at sites of BON, FPK, GWN, TBL, DRA and PSU. (**a**) Comparison of the trends of the *in-situ* measured LSTs and the spatiotemporally continuous all-weather LSTs at these six ground sites; (**b**) scatterplots of the original satellite-derived LSTs and the ground measurements; (**c**) scatterplots of the DIONEOF-reconstructed ideal, clear-sky LSTs (abbreviated as DINEOF R-ed LST in the figure) against the *in-situ* measured LSTs; and (**d**) scatterplots of the CDF matching-corrected real LSTs under cloudy sky conditions (abbreviated as CDF C-ed LST in the figure) against the *in-situ* measured LSTs.

**Fig. 6:**
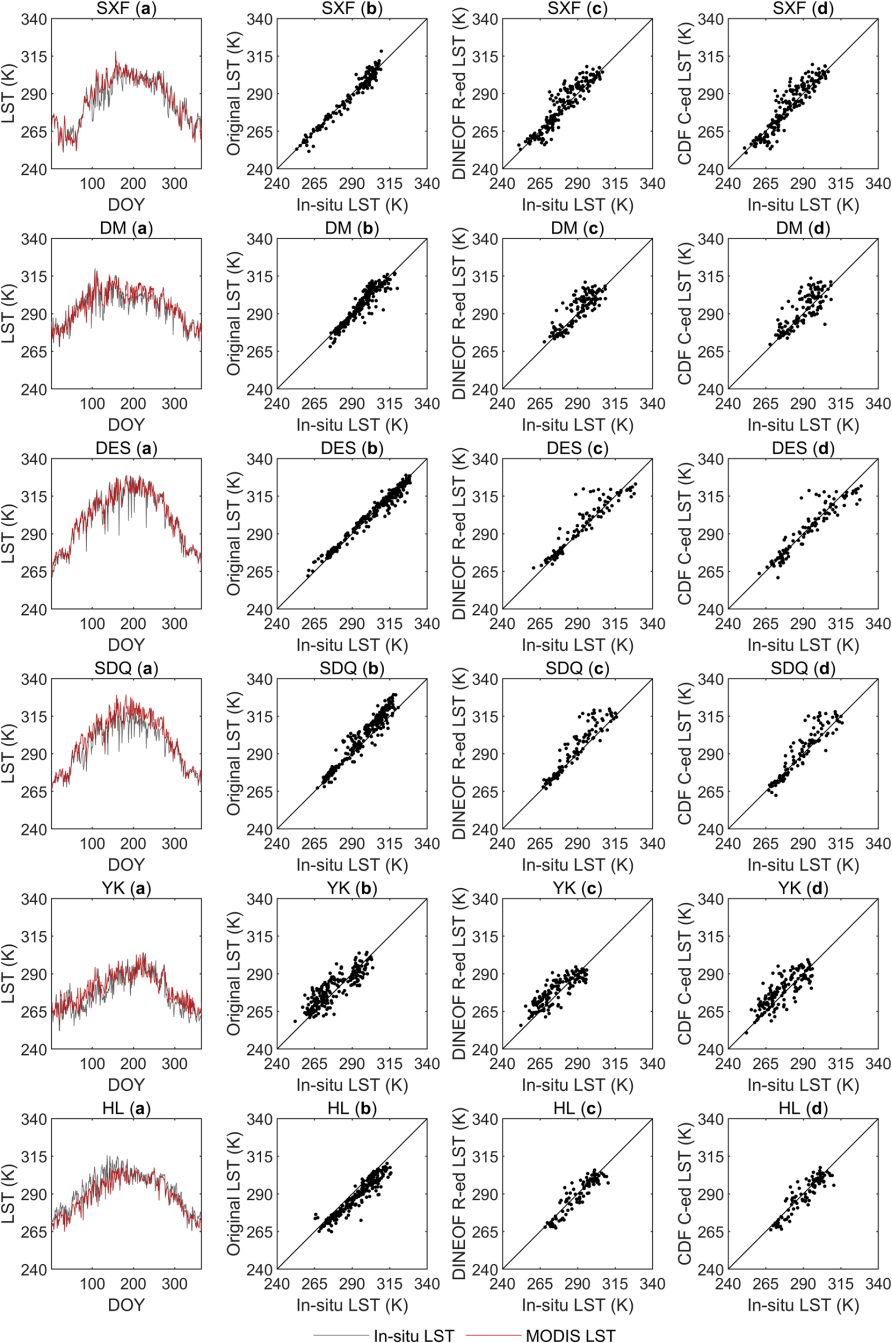
Comparison between the original satellite-derived LSTs and the reconstructed LSTs with the *in-situ* measured LSTs at sites of SXF, DM, DES, SDQ, YK and HL. (**a**) Comparison of the trends of the *in-situ* measured LSTs and the spatiotemporally continuous all-weather LSTs at these six ground sites; (**b**) scatterplots of the original satellite-derived LSTs and the ground measurements; (**c**) scatterplots of the DIONEOF-reconstructed ideal, clear-sky LSTs (abbreviated as DINEOF R-ed LST in the figure) against the *in-situ* measured LSTs; and (**d**) scatterplots of the CDF matching-corrected real LSTs under cloudy sky conditions (abbreviated as CDF C-ed LST in the figure) against the *in-situ* measured LSTs.

Figures 5 and 6 shows the comparisons between the original satellite-derived LSTs and the reconstructed LSTs with the *in-situ* measured LST. Figures 5 (a) and 6(a) shows the comparisons between the trends of the *in-situ* measured LSTs and the spatiotemporally continuous all-weather LSTs at twelve ground sites in 2019. In Figs. 5(b) and 6(b), scatterplots of the original satellite-derived LSTs against the ground measurements from twelve ground sites are shown. Figures 5(c), 6(c) and Figs. 5(d), 6(d) show scatterplots of the ideal, clear-sky LSTs reconstructed by the DINEOF method and the real LSTs under cloudy conditions corrected by the CDF matching method against the *in-situ* measured LSTs respectively. The corresponding statistical metrics of Figs. 5 and 6 are shown in Table 4. In Figs. 5(b) and 6(b), at most sites, the satellite-derived LSTs were consistent with the ground measurements, where R ranged between 0.867 and 0.986, the minimum bias was 0.001 K, the maximum bias was −5.595 K, and the RMSE was between 3.243 K and 6.754 K. Although each site had missing satellite observations on different days in 2019 and some sites had missing data for more than 200 days, all missing data were effectively filled in through the DINEOF method and the CDF matching method, completely reconstructing the LST time series data of each site. All of the DINEOF-reconstructed LSTs exhibited errors that were comparable to the errors of the satellite observations, where R ranged from 0.798 and 0.938, the smallest Bias was −0.570 K, the largest bias was 4.159 K, and the RMSE was between 4.326 K and 8.443 K. It can be seen by comparing Figs. 5(c), 6(c) and Figs. 5(d), 6(d) that after correcting the ideal, clear-sky LSTs produced by the CDF matching-based method, the three statistical metrics (the Bias, RMSE and R) had different degrees of improvement at the FPK, GWN, TBL, DRA, SXF, DM, DES, SDQ and YK sites. The Bias values at the FPK, GWN, TBL, DRA, SXF, DM, DES, SDQ and YK sites all decreased by 0.071–2.534 K; the RMSEs at the GWN, TBL, DRA, DM, DES and SDQ sites decreased by 0.071–1.434 K; and the R values at the TBL, DRA, SXF and DM sites increased by 0.001–0.042. The Biases and RMSEs at the BON, PSU and HL sites increased slightly, and the R values decreased slightly. The reason for this may be that the 0.1° spatial resolution ERA5-Land LST dataset was resampled to 0.05° to match the spatial resolution of MODIS LST, but the resampling process could not provide sufficient detailed information and may have introduced uncertainty to the CDF matching-based correction process. In addition, Figs. 5(a) and 6(a) exhibits good consistency between the reconstructed spatiotemporally continuous all-weather MODIS LSTs and the ground-measured LSTs. Overall, the combination of the DINEOF reconstruction method and the CDF matching-based correction method is an effective approach for MODIS LST reconstruction.

**Table 4:** Statistical metrics corresponding to Figs. 5 and 6.

	Bias (K)	RMSE (K)	R	n		Bias (K)	RMSE (K)	R	n
BON (a)	0.361	4.911	0.926	365	SXF (a)	0.446	4.880	0.952	365
BON (b)	−0.539	4.348	0.936	159	SXF (b)	−0.695	3.311	0.979	165
BON (c)	1.045	5.300	0.914	206	SXF (c)	1.386	5.865	0.926	200
BON (d)	1.376	5.730	0.910	206	SXF (d)	0.905	6.007	0.927	200
FPK (a)	0.520	4.769	0.965	365	DM (a)	0.760	4.960	0.901	365
FPK (b)	−0.392	3.472	0.983	192	DM (b)	0.001	4.151	0.932	241
FPK (c)	1.527	5.878	0.938	173	DM (c)	2.580	7.228	0.798	124
FPK (d)	0.361	6.219	0.933	173	DM (d)	2.231	6.239	0.840	124
GWN (a)	−3.465	5.382	0.904	365	DES (a)	0.405	4.398	0.972	365
GWN (b)	−4.534	5.300	0.941	184	DES (b)	−0.241	3.243	0.986	255
GWN (c)	−2.367	5.465	0.854	181	DES (c)	1.903	6.310	0.937	110
GWN (d)	−1.470	5.394	0.834	181	DES (d)	−0.270	6.204	0.933	110
TBL (a)	0.455	6.232	0.903	365	SDQ (a)	4.031	6.356	0.960	365
TBL (b)	−1.958	4.030	0.962	216	SDQ (b)	3.980	5.964	0.968	260
TBL (c)	3.920	8.443	0.839	149	SDQ (c)	4.159	7.237	0.933	105
TBL (d)	−1.386	7.009	0.869	149	SDQ (d)	1.880	6.435	0.925	105
DRA (a)	−4.528	6.789	0.957	365	YK (a)	2.214	6.591	0.862	365
DRA (b)	−5.595	6.754	0.967	266	YK (b)	1.896	6.654	0.867	215
DRA (c)	−1.624	6.885	0.905	99	YK (c)	2.670	6.500	0.854	150
DRA (d)	0.025	6.617	0.907	99	YK (d)	2.599	7.299	0.811	150
PSU (a)	−1.312	4.142	0.934	365	HL (a)	−3.504	5.464	0.939	365
PSU (b)	−2.309	3.881	0.961	157	HL (b)	−4.352	5.788	0.951	264
PSU (c)	−0.570	4.326	0.926	208	HL (c)	−1.287	4.510	0.930	101
PSU (d)	−0.816	4.503	0.923	208	HL (d)	−1.310	4.830	0.922	101

It is worth noting that in 2019, most of the twelve ground sites had more than 100 days of missed MODIS LST data. In particular, the BON, PSU and SXF sites lacked effective satellite observations for more than 200 days throughout the year, which illustrated the importance of LST reconstruction in practical applications.

### Cross validation against the ERA5-Land estimates

**Fig. 7:**
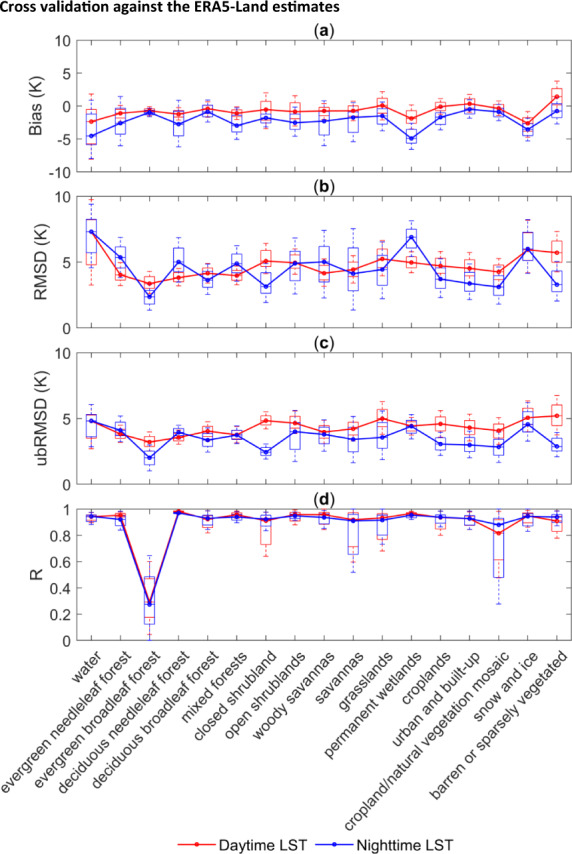
Boxplots of the variations in the performance metrics for different land cover types: (**a**) Biases, (**b**) RMSDs, (**c**) ubRMSDs and (**d**) R values.

**Fig. 8:**
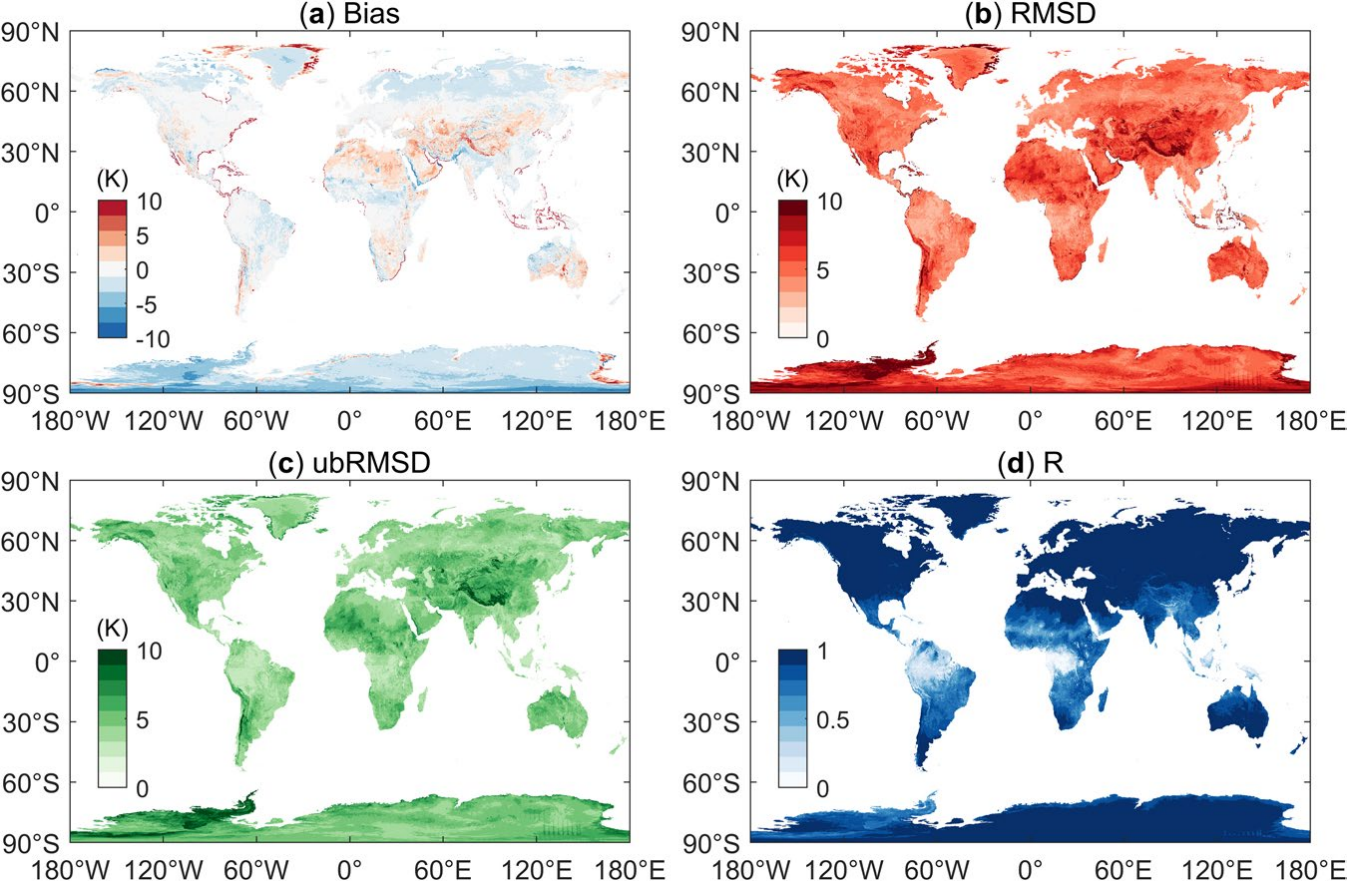
Global variations in the performance metrics: (**a**) Bias, (**b**) RMSD, (**c**) ubRMSD and (**d**) R.

After cross validating the LSTs corrected by the CDF matching-based method using the ERA5-Land LSTs at a global scale, we expressed the uncertainties by means of the Biases, RMSDs, ubRMSDs and R values for different land cover types, as shown in the boxplots in Fig. 7. The statistical metrics for the comparison between the daytime LSTs and the nighttime LSTs are given separately. In Fig. 8, the global spatial distribution of the Bias, RMSD, ubRMSD and R values of the corrected LSTs is shown. Figures 7 and 8 demonstrate that for most regions and land cover types in the world, the corrected LSTs exhibited a general consistency with the ERA5-Land estimates.

As shown in Fig. 7, the Biases and ubRMSDs at night were generally lower than those during the day for all land cover types. This may be because LSTs have larger spatial and temporal variabilities during the daytime, resulting in the final reconstruction accuracy being lower than that at night. The largest differences between the corrected MODIS LSTs and the ERA5-Land LSTs were observed over the water areas, where the largest mean RMSD was 7.308 K. The smallest R values between the corrected MODIS LSTs and the ERA5-Land LSTs were observed in the evergreen broadleaf forest-covered areas. In these evergreen broadleaf forest-covered areas, the lush foliage of trees hinders the reflection and emission of radiation from ground objects into space; in contrast, the transpiration of plants results in much water vapor, which also affects the radiation reflected and emitted by the ground. These are all important reasons that affect the observations of the satellite sensor when obtaining LSTs. The lack of effective satellite observations will inevitably affect the final reconstruction accuracy, and the correlation could be very low due to the shrinkage in the variability of LSTs. From the spatial distributions of the statistical metrics, the average Bias was −0.805 K, the average RMSD was 5.636 K, the average ubRMSD was 4.810 K and the average R was 0.863. In the equatorial area, the correlation coefficient R was significantly lower than that in other regions, which may be due to the perennial cloud coverage in these areas, as this results in proportions of missing data that are too high. In follow-up studies, the algorithm needs to be improved to solve these related problems.

## Usage Notes

In this study, we provided two sets of global spatiotemporally continuous MODIS LST data from 2002 to 2020 for various applications and studies. Users can freely choose the global spatiotemporally continuous ideal, clear-sky MODIS LST dataset or the global spatiotemporally continuous all-weather dynamic MODIS LST dataset according to their specific research directions. All the data are stored in hdf5 format as unsigned 8-bit or 16-bit integers with one file per day, and users can use MATLAB, Python, IDL, etc. to read and manipulate the data. It should be noted that the data extracted from the SDSs must be multiplied by their corresponding scale factors (in Table 1).

Notably, the uncertainties were higher than those of the final reconstructed MODIS LSTs in some areas covered by clouds year round (such as the equatorial area), and these data should be used with caution. The dataset will be updated in the future when new data become available.

## Data Availability

All the codes used in this study to construct the dataset were written in the MATLAB language and will be openly available at https://github.com/YuPeiHPU/ReconstructGlobalMODIS-LST.git under GNU Affero General Public License v3.0 after this work is accepted. The code used to implement the DINEOF method is openly shared by Azcarate^60^ at https://github.com/aida-alvera/DINEOF.git.
